# The Predictive Value of Plasma Brain Natriuretic Peptide and Galectin-3 in Elderly Patients Admitted for Heart Failure †

**DOI:** 10.3390/diseases6040088

**Published:** 2018-09-27

**Authors:** Marzia Testa, Gian Luca Rosso, Cinzia Ferreri, Mauro Feola

**Affiliations:** 1 Cardiovascular Rehabilitation-Heart Failure Unit Ospedale SS Trinità Fossano, 12045 Fossano, Italy; 2 Emergency 118 ASL CN1 Cuneo, 12100 Cuneo, Italy; gianluca.rosso@aslcn1.it; 3 School of Geriatry Università degli Studi Torino, 10134 Turin, Italy; cinzia.ferreri@gmail.com; 4 Division of Cardiology, Ospedale Regina Montis Regalis, Strada del Rocchetto 99, 12084 Mondovì, Italy

**Keywords:** galectin-3, B type-natriuretic peptide, heart failure, elderly

## Abstract

Galectin-3 is demonstrated to be a robust independent marker of cardiovascular mid-term (18-month) outcomes in heart failure (HF) patients. The aim of this study was to analyze the value of a pre-discharged determination of galectin-3 alone and with brain natriuretic peptide (BNP) in predicting mid-term outcomes in elderly patients (>70 years old) discharged after an acute decompensated HF (ADHF) episode. Methods: all elderly (≥70 years old) HF subjects discharged alive after an ADHF were enrolled. All patients underwent a determination of BNP and galectin-3, a 6-min walking test (6MWT), and an echocardiogram within 48 h of hospital discharge. Cardiac death, cardiac transplantation, and worsening heart failure requiring readmission to hospital were considered cardiovascular events. Results: 84 patients (63 males (75%), age 77.5 ± 5.9 years old) were analyzed (mean follow-up: 16.2 ± 12.3 months). During the follow-up, 45 events (53.6%) were scheduled (18 cardiac deaths, 27 re-hospitalizations for ADHF). HF patients who suffered an event demonstrated more impaired ventricular function (*p* = 0.04), higher value of BNP (*p* = 0.02), and Gal-3 at a pre-discharge evaluation (*p* = 0.05). By choosing adequate cut-off points (BNP ≥ 500 pg/mL and Gal-3 ≥ 17.6 ng/mL), the Kaplan–Meier curves depicted a powerful stratification using Galectin-3 > 17.6 ng/mL alone (log-rank 13.22; *p* = 0.0003), and by adding BNP + Gal-3, an even better result was obtained (log-rank 17.96; *p* < 0.00001). Conclusion: in an elderly population, by adding Gal-3 to BNP, a single pre-discharge strategy testing seemed to obtain a satisfactorily predictive value in alive HF patients discharged after an ADHF episode.

## 1. Introduction

Heart failure (HF) affects millions of patients and is the most common reason for hospitalization among the elderly; the length of hospitalization is usually >2 weeks in geriatric wards and readmissions are frequent [1]. Rates of heart failure hospitalization remain unacceptably high. Hospitalizations for HF are associated with discomfort for patients and caregiver, and incur economic costs [2]. Recently the IN-HF outcome study, an Italian nationwide registry, has proved that the 30-day mortality after discharging for an acute decompensated heart failure (ADHF) episode is 2.8% and hospital readmission 6.2% [3]. Older age, longer in-hospital stay, the necessity for inotrope use, and worsening NYHA class identify HF patients discharged home who are at the highest risk of death or readmission [4,5]. Moreover, the natriuretic peptides (NP) are now established serum markers not only for diagnosis, but also for prognosis in acute and chronic HF [6,7]. Different prognostic scores have been evaluated in the last few years [5,8] in order to better stratify patients’ mortality risk, but they still remain unable to predict the risk of rehospitalization [9,10]. A single value of a novel biomarker, galectin-3, in patients admitted for an ADHF has clearly demonstrated a powerful prognostic power in predicting mortality and rehospitalization at short or mid-term follow-up [11,12,13]. The aim of this study was to analyze the value of a single, predischarged determination of galectin-3 alone and in association with plasma brain natriuretic peptide (BNP) in predicting mid-term clinical outcome in elderly HF patients discharged after an ADHF episode.

## 2. Materials and Methods

This study, as an observational single-center study, included all consecutive HF subjects aged ≥70 years discharged alive after an acute episode of cardiac decompensation and enrolled in an out-patient clinic follow-up. Patients were classified as having HF according to the criteria commonly accepted in literature [14], namely the presence of two major criteria or one major criterion and two minor criteria according to the Framingham score and a NYHA functional class II, III, or IV, due to an exacerbation of symptoms with at least one class deterioration. The presence of inadequate echo images, no adherence to the optimal medical therapy, and disagreement with the periodical follow-up were considered exclusion criteria. All patients underwent a clinical examination, a 12-lead electrocardiogram, serum determination of BNP and Galectin-3 at discharge, a 6-min walking test, and a transthoracic echocardiogram within 48 h of hospital discharge. The therapy prescribed in those patients included angiotensin-converting enzyme inhibitors (enalapril, ramipril), angiotensin receptor blockade (valsartan) in case of enalapril/ramipril intolerance, beta-blockers (bisoprolol), digoxin, loop diuretic, and spironolactone at low dose. For beta-blockers, angiotensin-converting enzyme inhibitors and angiotensin receptor blockade, the patients’ maximum tolerated dose was used, after an adequate titration period.

Echocardiograms were performed with a Vivid 7 computed sonography system (GE Medical Systems, Waukesha, WI, USA) according to the recommendations of the American Society of Echocardiography [15]. Two-dimensional apical two- and four-chamber views were used for volume measurements; left ventricular ejection fraction (LVEF) was calculated with a modified Simpson method using biplane apical (two- and four-chamber) views. The left ventricle (LV) end-diastolic volume and the LV end-systolic volume were recorded. All the echo examinations were performed by expert operators blinded to the results of the BNP assay; the intra-observer variability in the evaluation of LVEF was found to be <5%. Echocardiographic measurements, including LV end-diastolic diameter and the diastolic thickness of the ventricular septum and the posterior LV wall, were determined according to the American Society of Echocardiography [16] recommendations. Systolic dysfunction was defined as a level of LVEF < 50%. The definition of restrictive filling pattern (grade 3) was a predefined modification of classifications used in prior studies: E/A ≥ 2, DT ≤ 150 ms, S/D ratio < 1, and AR > 35 cm/s. All these criteria should be verified to define the restrictive filling pattern. The other diastolic filling patterns were classified as: grade 1 (abnormal relaxation) with E/A < 1 and a DT > 240 ms; grade 2 (pseudo-normal) with E/A between 0.75 and 1.5, DT between 160 and 240 ms, and finally E/Ea > 1.5 [16]. The Doppler sample was set at 1–2 mm under the free edges of the mitral valve using the apical four-chamber projection; an average of five beats was considered. In patients suffering from atrial fibrillation at the time of the echocardiogram, the diastolic function was classified as: restrictive pattern (DT ≤ 150 ms) or indeterminate (DT > 150 ms). The presence of this diastolic pattern with an LVEF > 50% was defined as an isolated diastolic dysfunction. The tricuspid annular plane systolic excursion was measured in a four-chamber view by placing the 2-D cursor at the tricuspid lateral annulus and measuring the distance of systolic annular RV excursion along a longitudinal line defining the end of systole as the end of the T-wave in the electrocardiogram. Systolic right ventricular (or pulmonary artery) pressure was calculated using the modified Bernoulli equation: PAP = 4 × (tricuspid systolic jet) + 10 mmHg (estimated right atrial pressure).

Whole blood was obtained from subjects via standard venipuncture just before discharging when patients were considered stabilized after an acute HF admission. Serum was isolated within 60 min of sampling, shipped overnight, refrigerated, and stored at −70 °C until the time of testing. Galectin-3 was analyzed using an ELISA (BG Medicine, Foxboro, MA, USA). This assay has a lower limit of detection of 1.13 mg/L and demonstrated no cross-reactivity with other galectins or collagens [17]. The total precision of the assay at concentrations of 17.6 and 26.3 mg/L was 5.1% and 4.2%, respectively. The bedside Triage B type natriuretic fluorescence immunoassay (Alere Diagnostics, PSan Diego, CA, USA) was also used in all populations studied. The Triage Meter was used to measure BNP concentration by detecting a fluorescent emission that reproduces the amount of BNP in the blood. A total of 1 mL of whole blood was added to the disposable device, then the cells were filtered and separated from the plasma with BNP, which entered a reaction chamber, containing fluorescent BNP antibodies. After a 2-min incubation, the BNP–antibody mixture migrated to an area containing immobilized antibodies and remained fixed. The unbound fluorescent antibodies were washed away by the excess sample fluid. Then the Triage Meter measured the fluorescent intensity of the BNP assay area. The assay results were complete in 15 min. The performance characteristics of the test were: assay range 5 to 5000 pg/mL, total CV 9.2% to 11.4%.

The 6MWT (six-minute walking test) was performed at admission and discharge according to the ATS Statement of the American Thoracic Society [18]. HF patients able to walk underwent 6MWT if they did not meet the exclusion criteria (unstable angina and myocardial infarction during the previous month, resting heart rate > 120; systolic blood pressure > 180 mmHg, or diastolic blood pressure > 100 mmHg).

The Barthel Index was calculated both at admission and discharge. This is a scoring technique developed in 1965 and later modified by Granger et al. [19] that measures a patient’s performance in 10 activities of daily life. These activities can be divided into a group that is related to self-care (feeding, grooming, bathing, dressing, bowel and bladder care, and toilet use) and a group related to mobility (ambulation, transfers, and stair climbing). The maximum score is 100, if 5-point increments are used, indicating that the patient is fully independent in physical functioning. The lowest score is 0, representing a totally dependent bedridden state.

Likewise, the ADL (activity of daily living) scale [20] assesses the number of preserved basic daily activities out of a maximum of six functions, whereas the IADL scale [21] (instrumental activities of daily living) studies the subjects’ autonomy in eight instrumental activities of daily life needed to live independently at home.

Cardiac death, cardiac transplantation, and worsening of HF requiring readmission to hospital were considered cardiovascular events. Data regarding the occurrence of cardiovascular events were collected from multiple sources in all patients (follow-up consultations or phone calls). The study protocol conforms to the ethical guidelines of the 1975 Declaration of Helsinki; informed consent was required and signed by every patient and the study protocol was approved by a local ethics committee.

## 3. Statistical Analysis

Descriptive statistics were used to report the prevalence of various parameters. Categorical data were presented as numbers (percent), and continuous data were presented as mean ± standard deviation (SD) for normally distributed variables. The Shapiro–Wilk test was used to evaluate whether or not the distribution of the variables was normal. The mean values of any two groups were compared using the Student’s *t*-test and the means of more than two groups were assessed using analysis of variance followed by the Bonferroni multiple-comparison test. The Pearson χ^2^ test and the Fischer exact test were used for comparing categorical variables (NYHA class, cardiovascular events). The Spearman rank-order correlation coefficient (ρ) was used to measure the strength and direction of association between galectin-3 and glomerular filtrate and between galectin-3 and BNP. The outcome variables were coded 1 for hospitalization for HF or dead patients discharged after an ADHF episode, and 0 otherwise. The analysis was undertaken as follows: univariate logistic regression models were used to identify factors associated with events (as previously coded). The factors that were associated with the outcome measure with a *p*-value < 0.20 in the univariate analysis were then included in a multivariate logistic regression model. Event-free survival was estimated using the Kaplan–Meier method and curves were compared with the log-rank test. A *p*-value < 0.05 was considered significant. All statistical calculations were performed on STATA software (version 11.0 STATA Corporation, College Station, TX, USA).

## 4. Results

Eighty-four patients [63 males (75%), age 77.5 ± 5.9; range 65–90] were discharged alive after a new diagnosis of HF or for acute decompensation in chronic HF and were requested to enter the study, and after signing an informed consent, were enrolled. The etiology of HF was interpreted as: 35 ischemic (41.7%) treated with a CABG in 14 cases and PTCA in 12 (both procedures in 9 cases), 38 valvular cardiomyopathy (45.2%), 12.5% cardiomyopathy and 0.6% other causes; 36.9% had diabetes and 29.8% had an ICD (implantable cardioverter defibrillator). The main characteristics of the population examined are reported in Table 1. During the follow-up (16.2 ± 12.3 months), 45 events (53.6%) were scheduled (18 cardiac deaths and 27 rehospitalizations for HF).

The main differences between the Event-group versus the No-Event group are described in Table 2. Patients with a worse clinical outcome had a more severe impairment in LV systolic function (34.1 ± 2.5% vs. 41.6 ± 2.7%, *p* = 0.02) without a significant LV enlargement or a right ventricular involvement and a worse renal function (1.6 ± 0.2 mg/dL Event group vs. 1.3 ± 0.1 mg/dL No-Event group, *p* = 0.08). Moreover, in the Event group, a higher value of BNP (1431.9 ± 178.1 pg/mL vs. 844.5 ± 160.8 pg/mL, *p* = 0.02) and galectin-3 (28.1 ± 2.2 ng/mL vs. 21.7 ± 2.4 ng/mL, *p* = 0.05) emerged. A statistical difference in terms of medical therapy did not emerge between the two groups (Table 2).

A negative Spearman coefficient (ρ) of 0.50 indicated a moderate positive correlation between the galectin-3 value and creatinine (*p* < 0.00001), while a negative correlation (ρ = −0.36, *p* = 0.01) between galectin-3 and the Barthel Index and ADL was detected (ρ = −0.36, *p* = 0.01 and ρ = −0.22, *p* = 0.1, respectively). 

For univariate and multivariate analysis, the value of galectin-3 was seen to be significantly associated with total events (death and rehospitalization for HF). In fact, for a cut-off value of galectin-3 > 17.6 ng/mL, the risk of a total event was found to be three times higher (OR 2.87, *p* = 0.022), independent of age (OR 2.89, *p* = 0.022) and diabetes (OR 2.56, *p* = 0.045). The predictive value of galectin-3 > 17.6 ng/mL seemed to be even stronger when associated with a value of BNP > 500 pg/mL (OR 3.54, *p* = 0.007). For multivariate analysis, the association of galectin-3 > 17.6 ng/mL and BNP > 500 pg/mL showed a significant correlation with a worse clinical prognosis, independent of renal function (OR 2.99, *p* = 0.025), LVEF (OR 2.98, *p* = 0.042), age (OR 3.56, *p* = 0.007), diabetes (OR 3.61, *p* = 0.009), and functional parameters measured with the Barthel Index (OR 5.43, *p* = 0.01).

The predictive value of galectin-3 was also demonstrated for mortality, both when considered alone (OR 4.43, *p* = 0.028) or when combined with BNP values (OR 5.25, *p* = 0.009), and this predictive value was independent of age (OR 5.64, *p* = 0.007), creatinine (OR 5.26, *p* = 0.011), diabetes (OR 4.57, *p* = 0.01), and LVEF (OR 6.20, *p* = 0.021).

In this study, galectin-3 was not shown to be a predictive parameter of readmission to hospital for HF, neither when considered alone (OR 1.24, *p* = 0.659), nor when combined with BNP (OR 1.29, *p* = 0.58) (Table 3 and Table 4).

**Figure 1:**
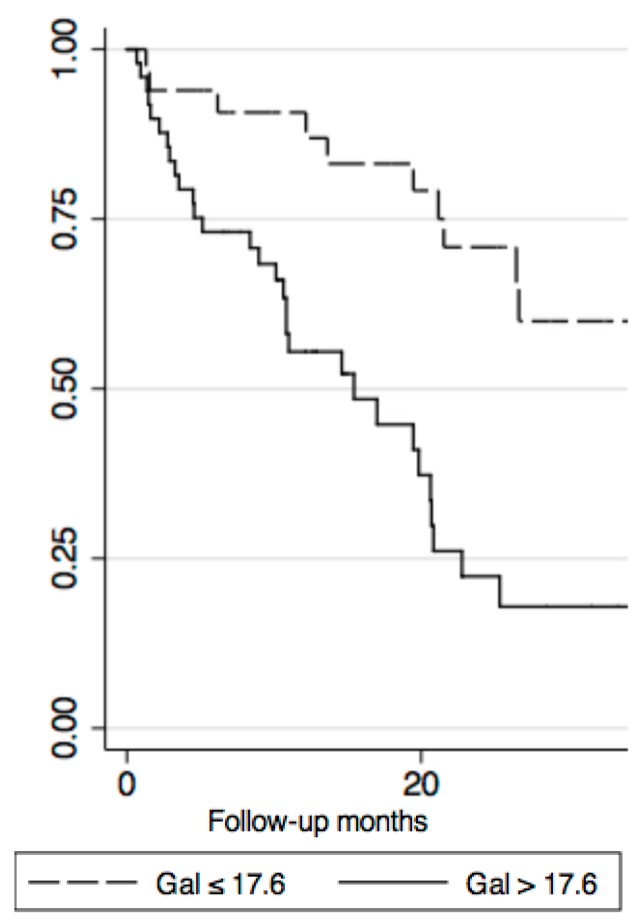
Kaplan–Meier curve with Galectin-3 > 17.6 ng/mL (log-rank 13.22; *p* = 0.0003).

**Figure 2:**
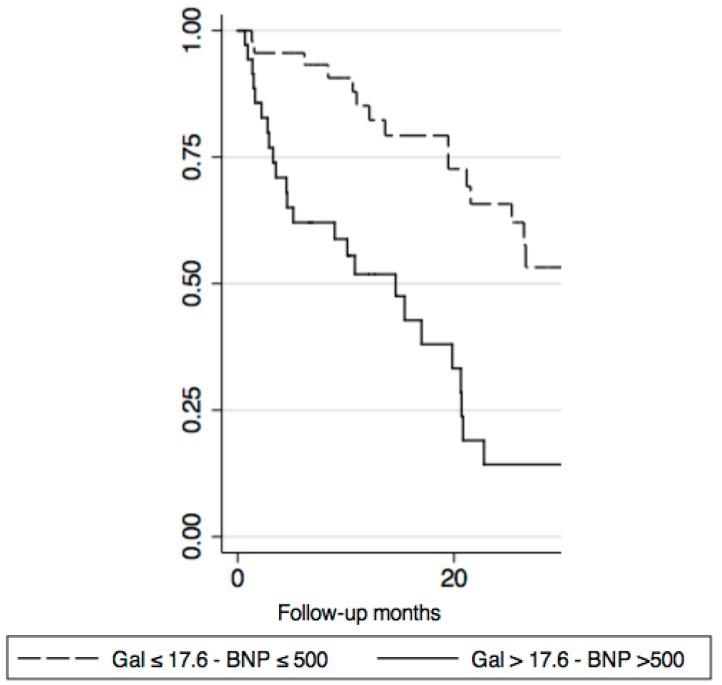
Kaplan–Meier curve with Galectin-3 > 17.6 ng/mL and BNP > 500 pg/mL (log-rank 17.96; *p* < 0.00001).

The Kaplan–Meier curves depicted a powerful stratification using a galectin-3 value > 17.6 ng/mL (log-rank 13.22; *p* = 0.0003) (Figure 1) and galectin-3 value > 17.6 ng/mL added to the plasma BNP level > 500 pg/mL (log rank 17.96; *p* < 0.00001) (Figure 2).

**Figure 3:**
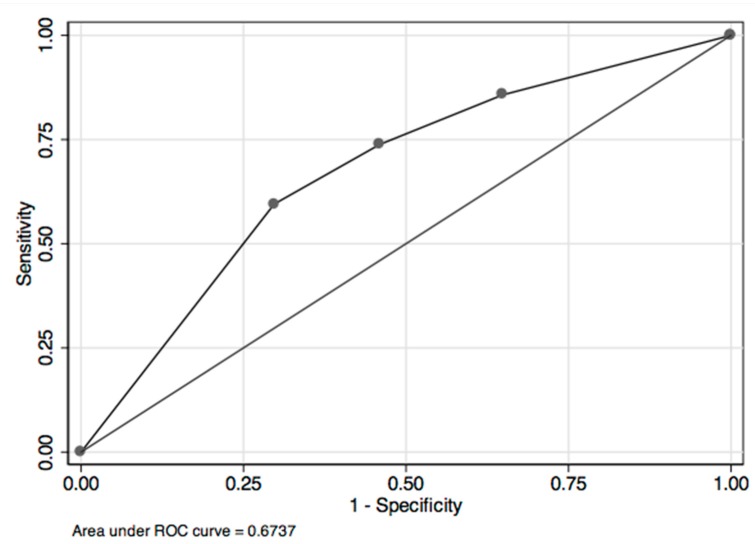
Roc Curve (AUC 0.67; *p* = 0.04) for galectin-3 value combined with BNP value.

A ROC curve (Figure 3) was obtained with an area under the curve of 0.67 (*p* = 0.04) when galectin-3 values were combined with BNP values, whereas the ROC curve obtained from a single value of galectin-3 or BNP was slightly less accurate (AUC 0.62, *p* = 0.01 and AUC 0.63, *p* = 0.007, respectively).

## 5. Discussion

The main finding of this clinical experience seems to be that a single pre-discharge determination of galectin-3 and BNP in hospitalized HF patients maintained their predictive value even in an elderly population (>70 years old). According to the huge number of elderly HF patients admitted to our hospitals [1,2,3], the persistence of efficacy of biomarkers in this real-world HF population might be of interest to physicians who usually take care of those patients.

Recently, the OPTIMIZE-HF [22] study described that a short length of hospitalization (4 days) is related to a higher rate of readmission within 30 days. This study highlights the fact that an early (1-week) outpatient clinical follow-up after discharge could help HF patients to have a lower probability of being readmitted within 30 days. Indeed, several prognostic parameters have been identified in order to easily stratify patients with a worse clinical outcome, including age, New York Heart Association (NYHA) class, renal function, arterial hypotension, and comorbidity such as atrial fibrillation, diabetes mellitus, and ischemic heart disease [4]. Moreover the natriuretic peptides (NP) are now established serum markers for diagnosis and prognosis in acute and chronic HF. 

Galectin-3 is a beta galactoside binding lectin, which has been demonstrated to be involved in cardiac fibrosis and ventricular remodeling [23,24]. In discharged HF patients, the combination of galectin-3 and NT-proBNP seemed to be the best predictor for short-term (60-day) mortality in the PRIDE study [11]. Furthermore, in the sub-study of COACH [13] galectin-3 was shown to be a robust independent marker of cardiovascular mid-term (18-month) outcomes in HF patients with a much stronger relevance in patients with preserved left ventricular ejection fraction (LVEF). In this study, in fact, galectin-3 lost its predictive value during multivariate analysis, when considered together with other risk factors such as reduced LVEF, diabetes, and renal impairment [14]. On the contrary, a low plasma level of galectin-3 (<11.8 ng/mL) proved to be an independent predictor (odds ratio 20.9; *p* = 0.003) for the absence of mortality and rehospitalization at short-term follow-up (6 months) [25]. Moreover, the pooled analysis of three clinical trials including 902 HF patients [26] demonstrated that HF patients with galectin-3 > 17.6 pg/mL had a greater risk (2.6–3 times) of being readmitted for ADHF from 30 to 120 days after discharge.

In discharged patients after an ADHF episode, a combined determination of galectin-3 and BNP seems to be the best predictor of short and mid-term mortality [27,28]. Indeed, as emerged in our recent experience, adding a single pre-discharge value of galectin-3 to BNP, a satisfactorily predictive value was obtained in predicting mid-term outcome in terms of mortality and re-hospitalization in “frequent flyers” (≥ 2 hospitalizations for HF/year) HF [29].

The previous experience on the predictive value of plasma biomarkers in elderly HF patients produced inconclusive results [22,30]. Indeed, while in OPTIMIZE-HF [22] patients over 65 years old, the BNP plasma value adequately predicted 1-year mortality and hospital readmission, the meta-analysis of Troughton et al., [30] clearly demonstrated how the survival benefit of a BNP-guided therapy post-discharge seemed to be effective only in younger (<75 years old) HF patients. Elderly HF patients more frequently experience comorbidity, frailty, multiple therapies, and significant cognitive impairment that have a notable impact on outcomes [31] independent of levels of biomarkers. In a recent review on plasma biomarkers, Correale et al. [32] stressed the fact that the role of biomarkers in elderly HF patients has been influenced by the presence of comorbidities. Comorbidities seemed to influence the response to NT-proBNP-guided therapy and may explain the lower efficacy of this approach in elderly patients [33].

In our single-center clinical study, we tried to add a piece of this complex puzzle by demonstrating that the single determination of galectin-3 together with BNP allowed a worse clinical outcome (death or hospital readmissions) at mid-long term (16 months) follow-up to be predicted. The predictive value of galectin-3, either alone (>17.6 ng/mL) or associated with BNP (respectively >17.6 ng/mL and >500 pg/mL), remained statistically significant independent of other risk factors (age, renal function, diabetes, and LVEF) (see Table 3 and Table 4). When analyzing mortality and re-hospitalization separately, galectin-3 and BNP maintained their predictive value only for mortality, lacking their power in predicting the risk of re-hospitalization. Considering the inconclusive results obtained by natriuretic peptide in terms of predictive value in the elderly HF population, the powerful stratification obtained using a pre-discharge single galectin-3 value > 17.6 ng/mL (log-rank 13.22; *p* = 0.0003) deserves consideration in the important area of plasma biomarkers. 

Furthermore, patients with a higher galectin-3 were seen to be frailer and functionally impaired (Barthel Index) and this highlights that a tailored clinical follow-up is mandatory in order to offer better support to these frail patients’ caregivers.

In conclusion, elderly patients (≥70 years old) with a higher pre-discharge value of galectin-3 (≥17.6 ng/mL) together with BNP (≥500 pg/mL) might be included into a rigid clinical follow-up, performed by a general practitioner or HF out-patient clinic, providing a strict control of body weight, hydration status, pharmacological adherence, and clinical status in order to prevent multiple readmissions and mortality.

### Limitations

The main limitation of this study was the small sample size observed, which might reduce the power of some consolidated prognostic parameters. Moreover, the sample size did not allow correct distinction between HFrE (HF reduced EF), HFpEF (HF preserved EF), and HFmrEF (HF mid range EF). The risk of multiple rehospitalization for HF is normally correlated to the severity of the cardiac condition, as well as other non-tested patient-related factors (social status, cognitive performance, and presence/absence of caregiver) that might significantly influence the rate of readmissions. Indeed, it was documented in the DIG TRIAL [34] that about 58% of the national variations in hospital readmission were correlated with socioeconomic factors. Moreover, in this study we analyzed only a single value of galectin3; it would be useful to evaluate another time-point biomarker in order to verify whether galectin-3 maintained its predictive value. In addition, this was an observational study only on acute decompensated HF where a meaningful conclusion could have been found if we used control group patients. In this HF elderly population, the presence of cancer or inflammatory disease might play a role as a confounder.

Finally, being a single-center study, it might be difficult to generalize these results to other elderly populations in different care settings. Our data, therefore, needs to be confirmed by larger sampling studies.

## Figures and Tables

**Table 1:** General characteristics of the population examined.

	*n* = 84
Age (years)	77.5 ± 5.9 (65–90)
Males	63 (75%)
CABG/PTCA	23 (27.4%)/21 (25%)
VALV	38 (45.2%)
CMP	10 (12.5%)
CAD	35 (41.7%)
ICD	25 (29.8%)
Diabetes	31 (36.9%)
Sinus Rhythm	38 (45.2%)
Creatinine (mg/dL)	1.5 ± 0.9
eGFR (mL/min/1.73 m^2^)	50.9 ± 21.7
Haemoglobin (g/dL)	12.6 ± 1.8
Sodium (mEq/L)	139.8 ± 3.3
BNP (pg/mL)	1156.8 ± 1108.7
Galectin-3 (ng/mL)	25.1 ± 15.1
LVEF (%)	37.8 ± 16.6
LVEF < 50%	60 (71.4%)
LVESD (mm)	47.3 ± 13.6
LVEDD (mm)	58.5 ± 11.2
PAPs (mmHg)	39.6 ± 9.9
NYHA admission	3.1 ± 0.8
NYHA discharge	2.2 ± 0.5
6-min WT admission (m)	248.4 ± 117.2
ADL admission	4.6 ± 2.1
ADL discharge	5.4 ± 1.2
IADL	1.4 ± 2.3
Barthel Index admission	83.8 ± 21.8
Barthel Index discharge	91.3 ± 15.4
Length of Hospital stay (days)	11.7 ± 5.5
Diuretics	82 (97.6%)
B-blockers	73 (86.9%)
ACE-Inibitor/ARB	42/34 (90.5%)
Spironolactone	40 (47.6%)

CABG: coronary artery bypass graft; PTCA: percutaneous transluminal coronary angioplasty; VALV: valvular disease; CMP: cardiomyopathy; CAD: coronary artery disease; ICD: implantable cardioverter defibrillator; eGFR: glomerular filtration rate; BNP: brain natriuretic peptide; LVEF: left ventricular ejection fraction; LVESD: left ventricular end systolic diameter; LVEDD: left ventricular end diastolic diameter; PAP: pulmonary artery pressure; NYHA: New York Heart Association; 6MWT: six minute walking test; ADL: activity daily living; IADL: instrumental activity daily living; ARB: angiotensin receptor blockade.

**Table 2:** Main characteristics between the Event group (death and rehospitalization for HF) and No-Event group.

	No-Event Group (39 pts)	Event Group (45 pts)	*p*
Age (years)	77.5 ± 0.8	77.6 ± 0.9	0.97
Sodium (mEq/L)	139.8 ± 0.6	139.7 ± 0.4	0.94
Haemoglobin (g/dL)	12.8 ± 0.3	12.5 ± 0.3	0.60
Creatinine (mg/dL)	1.3 ± 0.1	1.6 ± 0.2	0.08
eGFR (mL/min/1.73 m^2^)	53.3 ± 3.6	48.6 ± 3.5	0.35
BNP (pg/mL)	844.5 ± 160.8	1431.9 ± 178.1	0.02
Galectin-3 (ng/mL)	21.7 ± 2.4	28.1 ± 2.2	0.05
LVEF (%)	41.6 ± 2.7	34.1 ± 2.5	0.04
LVESD (mm)	44.5 ± 2.3	49.9 ± 2.2	0.098
LVEDD (mm)	56.7 ± 1.7	60.4 ± 2.1	0.17
TAPSE (mm)	15.8 ± 1.6	16.6 ± 0.8	0.65
PAP (mmHg)	38.0 ± 1.7	41.2 ± 1.7	0.19
6 MinWT admission (m) (*n* = 43)	261.6 ± 26.8	233.2 ± 23.2	0.43
6 MinWT discharge (m) (*n* = 42)	290.8 ± 20.1	261.0 ± 24.6	0.36
Length of Hospital stay (days)	11.5 ± 0.9	11.8 ± 0.8	0.78
Barthel Index admission	83.5 ± 4.5	84.1 ± 4.2	0.93
Barthel Index discharge	91.8 ± 3.5	90.9 ± 3.0	0.83
IADL	1.4 ± 0.4	1.4 ± 0.4	0.89
ADL at admission	4.8 ± 0.4	4.5 ± 0.4	0.53
ADL at discharge	5.4 ± 0.3	5.5 ± 0.3	0.68
Diabetes mellitus	12	19	0.95
Diuretics	38 (97.4%)	44 (97.7%)	0.5
B-blockers	34 (87.1%)	39 (86.6%)	0.79
ACE-Inibitor/ARB	17/19 (92.3%)	25/15 (88.8%)	0.8
Spironolactone	22 (56.4%)	18 (40%)	0.2

eGFR: glomerular filtration rate; BNP: brain natriuretic peptide; LVEF: left ventricular ejection fraction; LVESD: left ventricular end systolic diameter; LVEDD: left ventricular end diastolic diameter; TAPSE: tricuspid annular plane systolic excursion; PAP: pulmonary artery pressure; 6MWT: six-minute walking test; ADL: activity daily living; IADL: instrumental activity daily living; ARB: angiotensin receptor blockade.

**Table 3:** Logistic regression: Galectin-3 value > 17.6 ng/mL.

	Total Events		Death		Rehospitalization	
	(Death and Rehospitalization)					
	OR (95% CI)	*p*	OR (95% CI)	*p*	OR (95% CI)	*p*
Gal-3 > 17.6 ng/mL	2.87 (1.17–7.07)	0.022	4.43 (1.17–16.75)	0.028	1.24 (0.48–3.17)	0.659
Adjusted for age	2.89 (1.17–7.13)	0.022	4.35 (1.15–16.51)	0.031	1.27 (0.49–3.28)	0.622
Adjusted for LVEF	2.38 (0.92–6.16)	0.075	5.51 (1.12–27.18)	0.036	0.99 (0.37–2.72)	0.990
Adjusted for creatinine (eGFR)	2.35 (0.91–6.04)	0.077	4.17 (1.06–16.46)	0.041	1.07 (0.39–2.89)	0.890
Adjusted for diabetes	2.56 (1.02–6.41)	0.045	3.90 (1.00–15.22)	0.050	1.23 (0.46–3.29)	0.675
Adjusted for Barthel Index	3.76 (0.89–15.85)	0.071	0.98 (0.94–1.02)	0.333	1.73 (0.41–7.32)	0.454

**Table 4:** Logistic regression: Galectin-3 > 17.6 ng/mL + BNP > 500 pg/mL.

	Total Events		Death		Rehospitalization	
	(Death and Rehospitalization)					
	OR (95% CI)	*p*	OR (95% CI)	*p*	OR (95% CI)	*p*
Gal-3 > 17.6 ng/mL + BNP > 500 pg/mL	3.54 (1.40–8.91)	0.007	5.25 (1.52–18.10)	0.009	1.29 (0.51–3.26)	0.580
Adjusted for age	3.56 (1.41–9.01)	0.007	5.64 (1.59–19.88)	0.007	1.27 (0.50–3.21)	0.618
Adjusted for LVEF	2.98 (1.04–8.54)	0.042	6.20 (1.31–29.31)	0.021	1.08 (0.37–3.17)	0.892
Adjusted for creatinine (eGFR)	2.99 (1.15–7.80)	0.025	5.26 (1.47–18.85)	0.011	1.10 (0.41–2.92)	0.851
Adjusted for diabetes	3.61 (1.38–9.41)	0.009	4.57 (1.30–16.04)	0.018	1.47 (0.55–3.87)	0.441
Adjusted for Barthel Index	5.43 (1.49–19,7)	0.010	0.98 (0.93–1.02)	0.317	1.49 (0.42–5.21)	0.535
